# Gender differences in load carriage injuries of Australian army soldiers

**DOI:** 10.1186/s12891-016-1340-0

**Published:** 2016-11-25

**Authors:** Robin Marc Orr, Rodney Pope

**Affiliations:** Tactical Research Unit, Bond University, Gold Coast, QLD 4226 Australia

**Keywords:** Load carriage, Female soldier, Injuries, Pack march, Ruck march

## Abstract

**Background:**

With the removal of gender restrictions and the changing nature of warfare potentially increasing female soldier exposure to heavy military load carriage, the aim of this research was to determine relative risks and patterns of load carriage related injuries in female compared to male soldiers.

**Methods:**

The Australian Defence Force Occupational Health, Safety and Compensation Analysis and Reporting workplace injury database was searched to identify all reported load carriage injuries. Using key search terms, the narrative description fields were used as the search medium to identify records of interest. Population estimates of the female: male incident rate ratio (IRR) were calculated with ninety-five percent confidence interval (95% CI) around the population estimate of each IRR determined.

**Results:**

Female soldiers sustained 10% (*n* = 40) of the 401 reported injuries, with a female to male IRR of 1.02 (95% CI 0.74 to 1.41). The most common site of injury for both genders was the back (F: *n* = 11, 27%; M: *n* = 80, 22%), followed by the foot in female soldiers (*n* = 8, 20%) and the ankle (*n* = 60, 17%) in male soldiers. Fifteen percent (*n* = 6) of injuries in female soldiers and 6% (*n* = 23) of injuries in males were classified as Serious Personal Injuries (SPI) with the lower back the leading site for both genders (F: *n* = 3, 43%: M: *n* = 8, 29%). The injury risk ratio of SPI for female compared to male soldiers was 2.40 (95% CI 0.98 to 5.88).

**Conclusions:**

While both genders similarly have the lower back as the leading site of injury while carrying load, female soldiers have more injuries to the foot as the second leading site of injury, as opposed to ankle injuries in males. The typically smaller statures of female soldiers may have predisposed them to their observed higher risk of suffering SPI while carrying loads.

## Background

Soldiers are required to carry loads of up to 45 kg or more while performing combat tasks, often in unpredictable and hostile environments [1]. These loads, while vital for protection, sustainment and mission success [1], have been found to cause occupational injuries [2–4]. Furthermore, these occupational loads have been found to be heavier in combat arms trades [5] and are increasing in weight [6, 7]. With the recent removal of gender restrictions in combat arms trades for several military forces [8], there is potential in many nations for female soldiers to be more frequently exposed to heavy military load carriage and it is therefore timely to consider the injury risks that women may face in this role and compare these risks to those faced by men in this role, in order to determine whether any additional risk management strategies are indicated.

During load carriage tasks, female participants have typically been found to work at a higher percentage of their maximum aerobic capacity than their male counterparts when carrying the same absolute loads at the same intensity (e.g., same speed and gradient) [9–11]. These results are unsurprising, given the lower mean aerobic and anaerobic capacity, and lower absolute strength recorded in samples of military women when compared military men drawn from the same population [12]. However, it is appropriate to clearly acknowledge at this point that many of these mean gender differences affecting absolute work capacity result from gender-related differences in mean stature, social influences for sports and exercise participation, and other influential factors that can affect people of both genders. It follows, therefore, that the same issues will affect men of shorter stature and with less exercise history than other people, whether male or female. At a pragmatic, population level, though, such mean differences between the genders could substantially affect population levels of injury risk as women increasingly undertake load carriage roles. Any such influence on level of risk deserves proper assessment and management. However, it is important to note that the risk issues discussed herein do not only affect women; they also affect many men. Conversely, many women will possess sufficient stature and physical performance capacities to reduce their levels of risk at least to those experienced by the average male.

Findings from previous research are consistent with the mean gender differences introduced above. Female participants on average walk at a slower pace. They have also been found to take significantly longer than their male counterparts when able to self-determine the pace at which they complete a fixed load carriage task over a given distance [9]. This strategy allows women to maintain a workload that is as comfortable as possible and sustainable over time, an adaptive strategy that is commonly observed in soldiers carrying loads [13]. Holewijn, et al. [9] reported female participants worked at a mean 22% higher relative aerobic intensity level (determined as a proportion of individual VO_2_ max) than their male counterparts while performing a load carriage task at various controlled speeds in boots and wearing a fixed load of 12 kg in a waist pack. When both genders were required to work at the same relative aerobic intensity levels, the female participants on average walked at a slower pace (−0.7 to −0.8 km/h slower than male participants). These findings are supported by the results of studies conducted by Bhambhani and Maikala [11] and Harper, et al. [10]. Therefore, the research suggests that when required to maintain a given fixed, absolute task intensity, female participants typically must work at a higher percentage of their VO_2_ max than their male counterparts [9]. As well, when task intensity (e.g., speed of march) can be varied, they will choose to work at a similar preferred relative aerobic intensity (percentage of VO_2_ max) to that of their male counterparts, in turn typically resulting in longer event durations [9].

Load carriage tasks also typically elicit different biomechanical responses according to gender. On average, women are of shorter stature and walk with shorter stride lengths than males [14, 15]. Consequently, with gait speed the product of stride length and stride frequency [16, 17], the shorter stride lengths can require female participants to employ a higher mean stride frequency to maintain a given pace [14, 15]. In addition, it has been found that as the weights of the loads increase so too do the stride frequencies of female soldiers when attempting to maintain a given pace [14, 15]. A study by Martin and Nelson [15] examined the gait differences in male and female load carriers carrying loads in the range of 0 to 36 kg. They found female load carriers to have shorter stride lengths, on average, than male load carriers under all load conditions. However, the mean differences were only statistically significant when carrying heavier loads.

These findings raise questions regarding differences in load carriage injury rates and patterns between soldiers of each gender. Answering this question is important as it might lead to the identification of risk mitigation strategies. In addition, injuries caused by load carriage may decrease combat capability [1]. Therefore, the aim of this study was to determine the relative risks and patterns of injuries, including serious personal injuries, associated with contemporary military load carriage in female soldiers when compared to male soldiers.

## Methods

In this retrospective cohort study design, relevant occupational health and safety (OHS) incident data for all full-time Army personnel from the two calendar years 2009 and 2010 were identified and extracted from the Australian Defence Force Occupational Health, Safety and Compensation Analysis and Reporting (OHSCAR) database following departmental approval. The OHSCAR database is designed to capture all incident report forms submitted in the notification and reporting of Occupational Health and Safety (OHS) incidents that affect Australian Defence Force personnel and arise from the performance of Defence work [18]. The incident data were then paired with full-time Army population data, for the same years, for analyses.

Occupational Health and Safety incidents relating to injuries reported as having been sustained during load carriage events were extracted from the OHSCAR database. Load carriage events were defined as any activity where the soldier reported wearing webbing equipment, body armour or backpack, or where the specific activity at the time of the injury clearly indicated a load carriage activity.

Key search terms were used to search the narrative description fields of incident records in the OHSCAR database which related to incidents that had occurred in the period 01 January 2009 to 31 December 2010. Narrative description fields were used as the search medium to identify records of interest rather than the Type of Occurrence Classification System (TOOCS) data fields. This is because the TOOCS incident coding protocol codes incidents by the ‘most serious injury or disease sustained’ and the TOOCS ‘activity’ field has no specific load carriage codes.

The search terms used to identify relevant incident records from within the OHSCAR injury database were those commonly associated with contemporary military load carriage in the Australian Regular Army (ARA) and have been described in previous research [2]. These terms were; ‘pack’, ‘webbing’, ‘patrol’, ‘patrol order’, ‘march’, ‘marching order’, ‘route march’, ‘endurance march’, ‘Combat Fitness Assessment’, ‘CFA’, ‘load’, ‘load carriage’ and ‘carry’. Total numbers of ARA injury incidents reported over this period were also determined from the OHSCAR database.

Serious personal injuries (SPI) were defined as injuries that required immediate treatment (e.g., as an in-patient in a hospital) as defined by the Australian Department of Defence ‘event definition’ [18]. This injury severity classification was an entered field within TOOCS database with all other injuries classified as minor personal injuries (MPI) [18].

### Data extraction and analysis

Following manual cleaning of raw data, only records of injury incidents relating to contemporary military load carriage were retained. The manual cleaning consisted of reviewing each line of data individually and removing all incomplete data sets where variables were missing as well as any duplicate entries. Each line of data in the narrative description fields were also reviewed to ensure only activities specific to load carriage were retained All records unrelated to load carriage (e.g., the term ‘load’ used to describe the state of weapon readiness) were removed. As per previous research [2], the remaining incident records were then subjected to the inclusion and exclusion criteria detailed in Table 1 to derive the final data set for subsequent analysis.

**Table 1:** OHSCAR Injury data inclusion and exclusion criteria as described by Orr et al. (2014) [2]

Descriptor	Inclusion criteria	Exclusion criteria
Service Type	Australian Regular Army	Cadets Army Reserve Navy Air force Defence civilian
Incident Description	Injury first experienced during a load carriage event, immediately after a load carriage event, or the day following a load carriage event, with no indication of intervening activity	Load carriage identified but injury associated with other mechanisms (e.g., running)
Casualty type	Serious personal injury	Exposure^a^
	Incapacity	Dangerous occurrence^b^
	Minor injury	

^a^Exposure data were removed as this information is used to describe exposure to workplace hazards (like noise or radiation) that does not immediately or shortly afterward lead to incidents of injury meeting the inclusion criteria for casualty type

^b^Dangerous occurrence data were removed due to the data’s subjective nature and failure to meet inclusion criteria for casualty type

To determine gender differences in load carriage injury incident rates, the numbers of load carriage injuries suffered by soldiers of each gender across the two years of interest were first divided by the mean number of ARA soldiers of the respective gender who were serving during these years. These calculations yielded a two-year load carriage injury incident rate for each gender, which was then halved to yield a mean annual injury incident rate for soldiers of each respective gender. A population estimate of the female: male incident rate ratio (IRR), indicating the ratio of the injury incident rate in female soldiers to the injury incident rate in male soldiers, was calculated using the following formula [19]:

$$ \mathrm{I}\mathrm{R}\mathrm{R} = \left(\mathrm{female}\ \mathrm{injury}\ \mathrm{incident}\ \mathrm{rate}\right)/\left(\mathrm{male}\ \mathrm{injury}\ \mathrm{incident}\ \mathrm{rate}\right) $$

The numbers of female and male soldiers used in these calculations were based on ARA population figures reported in the Australian Defence Force Annual Reports for 2009–2010 [20].

The ninety-five percent confidence interval (95% CI) around the population estimate of each IRR was calculated as:

$$ 95\ \%\ \mathrm{C}\mathrm{I} = \exp\ \left( \ln \left[\mathrm{I}\mathrm{R}\mathrm{R}\right]\ \hbox{--}\ 1.96 \times \mathrm{S}\mathrm{E}\left( \ln \left[\mathrm{I}\mathrm{R}\mathrm{R}\right]\right)\right)\ \mathrm{t}\mathrm{o}\  \exp\ \left( \ln \left[\mathrm{I}\mathrm{R}\mathrm{R}\right] + 1.96 \times \mathrm{S}\mathrm{E}\left( \ln \left[\mathrm{I}\mathrm{R}\mathrm{R}\right]\right)\right) $$

where SE(ln[IRR]) = √ (1/[incident rate_female_] + 1/[incident rate_male_] – 1/n_female_ ‐ 1/n_male_) [19].

Further descriptive analyses were conducted to examine injury patterns evident in the data, based on counting the numbers of incidents that were associated with particular body parts, natures of injury, activity types, injury mechanisms, agencies of injuries, and other injury correlates. Calculations similar to those described above were conducted to derive IRR between genders for each of the five most common injury types and for serious personal injuries, all identified in the descriptive analyses.

Ethics approval for the research was granted by the Australian Defence Human Research Ethics Committee (Protocol 569–09), and the Behavioural and Social Sciences Research Ethics Committee of The University of Queensland (Protocol number: 2009001820). As the study utilised retrospective non-identifiable data, participant consent was not required by either ethics committee.

## Results

Records of 401 reported injuries associated with load carriage were identified from a total 1954 ARA injury records for the study period. The mean ARA population sizes in the study period comprised 2441 (10%) female soldiers and 22435 (90%) male soldiers. Commensurate with the gender ratio, 10% (*n* = 40) of the reported load carriage injuries were sustained by female soldiers and 90% (*n* = 361) by male soldiers, with the load carriage injury IRR for female soldiers compared to males being 1.02 (95% CI 0.74 to 1.41). The leading activities during which load carriage injuries were caused were the same in both genders being marching (♀ = 69%, ♂ = 59%), patrolling (♀ = 10%, ♂ = 13%), combat training (♀ = 10%, ♂ = 13%), and physical training (♀ = 8%, ♂ = 5%).

**Fig. 1:**
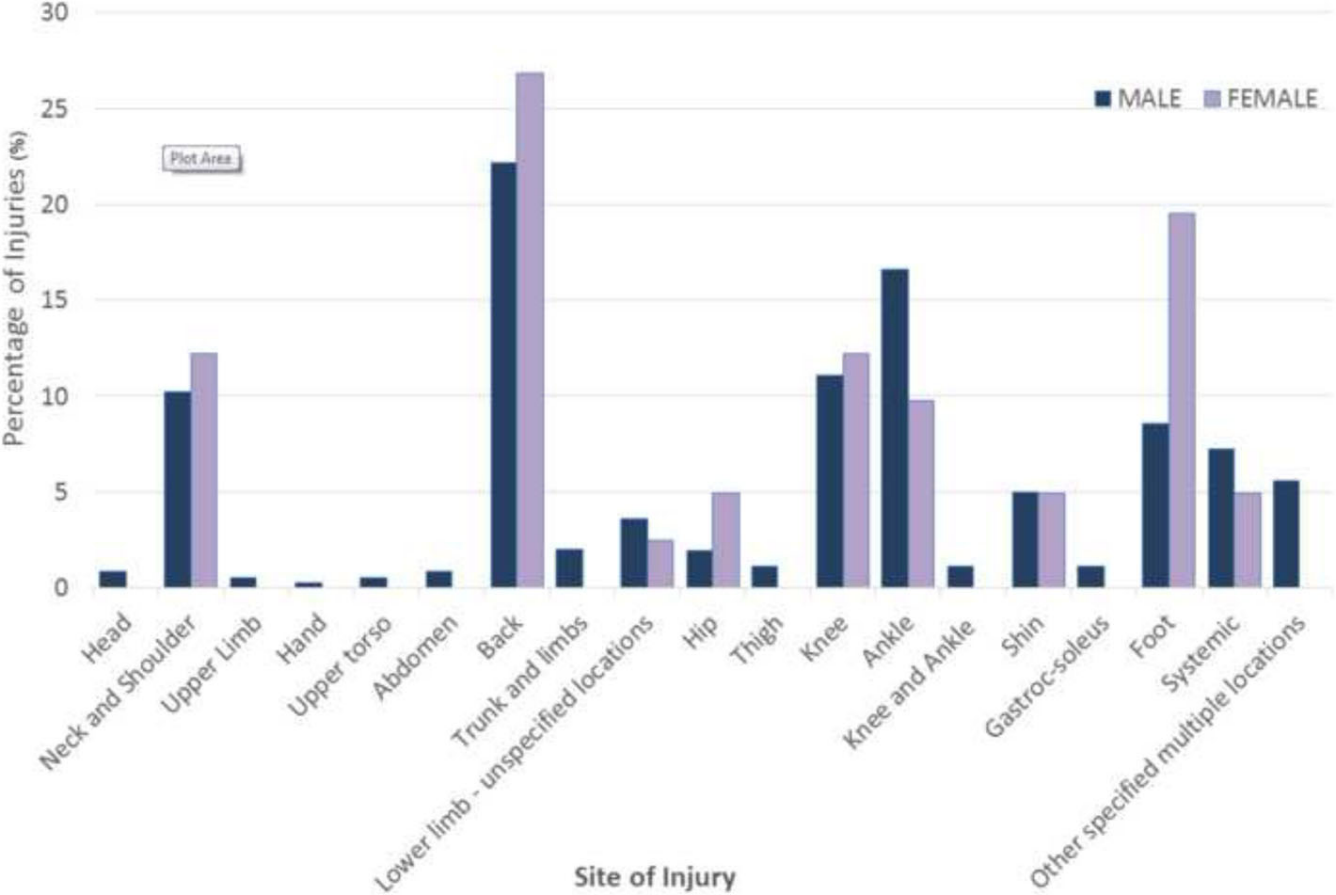
Body site distributions of load carriage injuries by gender

Figure 1 shows the most common site of load carriage injuries for both genders was the back (females: *n* = 11, 27% of female injuries; males: *n* = 80, 22% of male injuries). Completing the top five sites of injuries for female soldiers, the foot was the second most common site of injuries (*n* = 8, 20%), followed by the ‘neck and shoulder’ and knee (*n* = 5 each, 12% each) and ankle (*n* = 4, 10%). For male soldiers the ankle (*n* = 60, 17%), knee (*n* = 40, 11%), ‘neck and shoulder’ (*n* = 37, 10%) and foot (*n* = 31, 9%) were the next most common sites of injuries. The resulting IRR for female soldiers compared to male soldiers for each of these five most common load carriage injury types are as follows:

- Back injury IRR: 1.26 (95% CI 0.67 to 2.37)
- Foot injury IRR: 2.37 (95% CI 1.09 to 5.15)
- ‘Neck and shoulder’ injury IRR: 1.24 (95% CI 0.49 to 3.16)
- Knee injury IRR: 1.15 (95% CI 0.45 to 2.91)
- Ankle injury IRR: 0.61 (95% CI 0.22 to 1.69).

These IRR and their associated 95% CI indicate that, among the top five load carriage injury sites in female and male soldiers, female rates of load carriage injuries at each site were probably similar to male rates except in the case of foot injuries, where our best estimate indicates that female rates of load carriage injuries affecting the foot were more than twice those recorded for males.

Fifteen percent (*n* = 6) of load carriage injuries in female soldiers and 6% (*n* = 23) of load carriage injuries in males were classified as SPI, giving an IRR of load carriage-related SPI for female soldiers compared to male soldiers of 2.40 (95% CI 0.98 to 5.88). For both female and male soldiers, the lower back was the leading site for SPI (Females: *n* = 3, 43% of SPI: Males: *n* = 8, 29% of SPI). Systemic illness, through heat stress, was also a leading type of injury in male soldiers (*n* = 8, 29%) but not in females (*n* = 1, 14%), though clearly the small numbers of this type of SPI in the study sample may have obscured the rates of these types of SPI that actually occur over longer periods of time in the underlying population.

## Discussion

The aim of this study was to determine the relative risks and patterns of injuries, including SPI, associated with contemporary military load carriage in female soldiers when compared to male soldiers. As a preface to the discussion of the study’s findings, it is important to reiterate that the study was conducted in the period that immediately preceded the removal of gender restrictions in Australian Army combat arms trades. During the study period female soldiers in the Australian Army were not permitted to serve in combat arms corps of service (like infantry, engineers, artillery and armoured corps) [8]; corps known to carry heavier absolute loads [5]. The removal of this gender restriction [8], means that female soldiers may be exposed to heavier absolute loads should they serve in these trades. As such, the timing of this study is important as it means that the rates and patterns of load carriage injuries observed in the full-time female soldiers included in this study arose from carrying loads that would usually have weighed substantially less than the loads typically worn by soldiers in the combat arms trades both then and now [5]. Higher rates and levels of severity of injuries could be expected in female soldiers when heavier loads are carried. However, it should also be noted that many of the comparator male soldiers included in this study may not have been serving in combat arms roles, and so would also not usually have carried the substantially heavier loads typical of those roles. Though this limitation must be noted, the gender-based comparisons of injury rates and patterns conducted in the current study are likely to retain validity, albeit representing a conservative estimate of contemporary load carriage injury incidence rates in female soldiers.

The results of this study indicate that overall levels of load carriage injury risk between genders were not discernibly different in this period. However, our best estimate is that female soldiers had twice the level of risk experienced by male soldiers of suffering serious personal injuries and of suffering foot injuries, from carrying loads. The back was the leading body site of reported load carriage injuries in both male and female soldiers, and of reported serious injuries, in both female and male soldiers. Aside from the foot, the other most common injury sites in both female and male soldiers were the ankle, knee and ‘neck and shoulder’ complex, though the risks of these injury types were similar when female and male soldiers were compared. Systemic illness associated with heat stress was also reported to have occurred in numerous soldiers during the study period, mostly in male soldiers.

The results of the current study, identifying similar overall rates of load carriage injuries between genders, differ to results from studies comparing general injury rates in female and male army recruits. It is important to note that no comparative research could be found in a comprehensive search of the literature to have specifically investigated differences in load carriage injury rates between female and male soldiers. O'Connor [21] found that female army recruits had an overall higher incidence of injuries during recruit training. Similarly, in a study of serving soldiers, Strowbridge [22] found that female soldiers were more likely to sustain an injury than their male counterparts. They found that female soldiers, making up 5% of the unit’s population, sustained 8% of injuries over a three-year period. Interestingly, although female soldiers sustained significantly more injuries during military training, physical training and work than male soldiers, they sustained similar rates of injury to male soldiers when playing sport. This may reflect a greater capacity during sport to self-pace, in contrast to military and physical training and work, where workloads are often fixed.

One potential explanation for the differences in results between our study and others mentioned above is differences between the genders in the levels of exposure to the causal stimulus, load carriage. For example, in the study reported by Strowbridge [22] all injuries sustained were included, rather than just those from a specific task like load carriage. Any potential gender differences in injury rates reported in the current study may have been minimized by the limited participation of female soldiers in load carriage tasks relative to the levels of participation of male soldiers in load carriage tasks. As noted earlier, the current study was conducted prior to the integration of female soldiers into Arms Corps. Research within this population at that time has identified that male soldiers were exposed to operational load carriage tasks more often than female soldiers [5]. In addition, the nature of the tasks conducted and the loads carried varied. Female soldiers were less likely to conduct combat foot patrol tasks (requiring load carriage) and more likely to conduct administration tasks. As well, the absolute loads carried by male soldiers on operations were significantly heavier than loads typically carried by female soldiers [5]. Essentially, a lower rate of exposure to load carriage tasks and lighter absolute loads may have contributed to the lack of significant differences in overall injury rates between the genders in the current study even though the activities thought to cause these load carriage injuries were similar.

However, while absolute loads were higher in male personnel there were no statistically significant differences in relative loads between the two genders when the carrier’s body weight was accounted for (even though female soldiers carried slightly lighter relative loads of 43% of body weight when compared to male soldiers at 47% [5]). This suggests that while female soldiers were carrying lighter absolute loads, when the loads were considered in relation to their lighter body weight, the relative loads between genders were similar. This may raise concern in the future. As aforementioned, the removal of gender restrictions means that female soldiers may now be serving in corps carrying heavier load weights. This in turn may mean that while female soldiers, in general, would carry similar absolute loads to male soldiers, their relative loads would increase.

Despite similarities in overall load carriage injury rates observed in the current study, female soldiers were observed to be at a higher risk of suffering a serious personal injury related to load carriage. Three potential explanations include: carriage by some female soldiers in some instances of heavier total absolute or relative loads; a lower maximal aerobic capacity; and poor equipment fit. As noted above, previous research within this population suggests that the relative external loads typically carried by female (43% body weight) and male soldiers (47% body weight) may have been similar [5] However, the potentially higher levels of body fat mass in female soldiers may have further increased the total loads carried and the resultant lean muscle mass loading [23, 24]. In the study by Scott and Ramabhai [23], female soldiers who were carrying similar relative external loads (37% body weight) were found to work harder than the male soldiers. This higher work effort was considered to be due to greater body fat mass in the female participants who carried approximately 8 kg more body fat than their male counterparts. Thus, the female participants carried a mean load of 24 kg (37% body weight) plus 17 kg (fat mass), equating to a total passive load of 41 kg, whereas the male participants carried a mean load of 27 kg (37% of body weight) plus nine kilograms (fat mass), equating to a total passive load of 36 kg.

Absolute VO_2_ max has a strong correlation with load carriage task performance [25]. As noted earlier, female soldiers typically have a lower absolute maximal aerobic capacity than male soldiers [12]. These lower levels of fitness mean that female soldiers would typically have to work at a higher percentage of VO_2_ max than male soldiers to perform a given load carriage task [9]. In addition, lower levels of aerobic capacity are associated with an increased risk of injury [26]. As such, female soldiers may be more susceptible to severe load carriage injuries due to the higher level of metabolic stress they endure when performing a load carriage task.

Finally, the fit of load carriage equipment may have contributed to the heightened rate of serious load carriage injuries observed in female soldiers in the current study. Harper et al. [10] noted that female soldiers reported more problems with equipment fit than male soldiers, most notably shoulder straps and the stability of the rucksack. The authors [10] also noted that the effects of this poorer equipment fit meant female soldiers reported greater levels of pain, soreness and discomfort in the back region when carrying heavier loads and were hypothesized to contribute to the gender differences in pack marching injury rates between genders. In combination, a heavier load, lower general aerobic capacity and poorer equipment fit may have contributed to a greater incidence rate of serious load carriage injuries in female soldiers in the current study by creating additional stress on overloaded musculoskeletal structures and physiological systems.

In two previous studies [3, 27] investigating load carriage injuries over a single event, both with male only infantry populations, the body sites of injuries sustained during and immediately following the event were similar to the sites of injuries sustained by male participants in this study. In the study by Knapik et al. [3], back injuries, foot injuries and sprains to the foot, knee and ankle were the leading types of musculoskeletal injuries sustained. Conversely Reynolds et al. [27] reported the foot, back and knee to be the most common sites of musculoskeletal injuries during a load carriage event. Interestingly, neither of these two comparative studies reported any shoulder and neck complaints, injuries which were prominent in our study. A potential reason may be that the neck and shoulder complaints are more chronic in nature and occur over a more prolonged period as opposed to a single load carriage event as reported in these two studies. In addition, the mean Australian soldier loads reportedly carried over the period may have been a contributing factor. Where the infantry populations in the studies Knapik, et al. [3] and Reynolds, et al. [27] carried loads of 46 kg and 47 kg respectively, the Australian Army Infantry soldiers carried a mean general load of 49 kg and a mean operational load of over 60 kg over a two year (2009 and 2010) period [5]. This heavier load may have caused a greater compression force over the brachial plexus and surrounding musculoskeletal structures, leading to a higher incidence of neck and shoulder injuries. Potential difference in backpack design, shoulder strap width for example, may likewise have influenced the number of neck and shoulder injuries reported in male soldiers in this study.

The lower limbs in particular have been found to be the leading site of injury to military populations not on combat operations [28–30]. In some instances, injuries to the lower limbs have represented over 80% of reported injuries in a military population [30]. However, when serving in a combat theatre, the lower back typically presents as the leading site of injury [31, 32]. Potential reasons for this change in injury site may be the wearing of body armour and load carriage. Wearing body armour has been found to increase the physical demands of performing a given task [33] and as such it is not unexpected that it is associated with causing lower back injuries in military populations [34]. Likewise, load carriage has been found to change the shape of the spine [35, 36] and create cyclic stresses to the Vertebrae [37].

The finding in this study of a significantly greater frequency of foot injuries related to load carriage in female soldiers when compared to their male counterparts may be indicative of differential biomechanical loading of the musculo-skeletal system. Female soldiers have been found to have lower strength in ankle dorsiflexion, inversion and eversion than male soldiers from the same population [12]. Therefore, the fatigue resilience and ability to bear load afforded by the strength of these muscles which support the foot may be reduced. Greater frequency of foot injuries may also indicate an equipment fit issue, and this warrants further investigation. Ill-fitting boots have been noted as an issue for female soldiers [38]. Relative boot stiffness and the numbers of available boot width sizing in smaller boot sizes typically required by women are thought to contribute to problems with the boot-foot interface [38].

## Conclusion

The results of this study indicate that overall levels of load carriage injury risk for female soldiers and male soldiers were not discernibly different but that female soldiers had twice the level of risk experienced by male soldiers of suffering serious personal injuries and of suffering foot injuries, from carrying loads. As more women move into combat roles and carry heavier loads, it is likely that both overall rates of load carriage injuries and rates of serious load carriage injuries will increase in female soldiers. Key reasons for the gender-related differences in load carriage injury rates and patterns reported and predicted here at a population level are gender-related differences in mean stature, absolute aerobic capacity, body fat mass, muscle strength, and equipment fit. The fit of both loads to be carried and boots have historically been problematic for female soldiers. Considering this, similar problems are likely to be experienced by male soldiers of shorter stature and with lower levels of aerobic capacity and muscular strength. Conversely, women of taller stature, and with strong exercise histories and good muscle strength may experience lower rates of load carriage injuries than many men.

## Abbreviations

ARA: Australian regular army
CFA: Combat fitness assessment
CI: Confidence interval
IRR: Incident rate ratio
OHS: Occupational health and safety
OHSCAR: Occupational health, safety and compensation analysis and reporting
TOOCS: Type of occurrence classification system
